# 453. Neutralizing Antibody Responses to SARS-CoV-2 in Professional Soccer Players

**DOI:** 10.1093/ofid/ofab466.652

**Published:** 2021-12-04

**Authors:** Jorge Pagura, Clovis Arns de Cunha, Roberto Nishimura, Sergio Wey, André Pedrinelli, Sergio Cimerman, Andreia Picanço, Sérgio Freire, André Guerreiro, Edilson Thiele, Carlos Starling, Bráulio R G M Couto

**Affiliations:** 1 Comissão Científica da Confederação Brasileira de Futebol - CBF, São Paulo, Minas Gerais, Brazil; 2 Universidade Federal do Paraná; 3 Brazilian Society of Infectious Diseases, Sao Paulo, Brazil; 4 Sociedade Mineira de Infectologia - SMI, Belo Horizonte, Minas Gerais, Brazil; 5 Centro Universitário de Belo Horizonte - UniBH, Belo Horzonte, Minas Gerais, Brazil

## Abstract

**Background:**

The Brazilian Football Confederation (CBF) protocol to control the spread of COVID-19 among professional soccer players is based on four cornerstone measures: (1) Tracing all symptomatic and asymptomatic COVID-19 cases by clinical monitoring and nasal swab SARS-CoV-2 RT-PCR testing up to 3 days before the soccer games; (2) Respiratory isolation of all SARS-CoV-2 positive players for at least 10 days, regardless symptoms; (3) All player with clinical suspicion of COVID-19 were immediately quarantined; (4) If a player became SARS-CoV-2 positive after the game, the other players were allowed to play the next game, if they remained asymptomatic and SARS-CoV-2 RT-PCR negative. Understanding how antibody responses to SARS-CoV-2 evolve can provide insights into therapeutic and testing approaches for COVID-19. In the present study we profile the antibody responses of players up to nine months from a SARS-CoV-2 positive RT-PCR test.

**Methods:**

Serum samples were obtained from 955 soccer players, and analyzed at the same laboratory in São Paulo city, in the Hospital Israelita Albert Einstein. It was used the cPas Technology, the sVNT kit for detecting and measuring circulating neutralizing antibodies against the SARS-CoV-2 virus.

**Results:**

Neutralizing antibody was positive for 416 samples (416/955=44%; C.I. 95%= [40%; 47%]). From the 955 soccer players, 454 had RT-PCR+ previously, up to nine months until the neutralizing antibody tests. From this 454 players, 172 (38%) had neutralizing antibody below 20% (C.I. 95% = [34%; 42%]), 30 (7%) between 20% and 30% (C.I. 95% = [5%; 9%]), and e 252 (56%) above 30% (C.I. 95% =[51%; 60%]). Antibody responses to SARS-CoV-2 were significantly higher in individuals RT-PCR+ (Table 1). There was no difference between the neutralizing antibody responses status to SARS-CoV-2 and the time between the RT-PCR+ and the neutralizing antibody test (p-value = 0.423; Figures 1 and 2, Table 2).

Table 1. Neutralizing antibody responses to SARS-CoV-2.

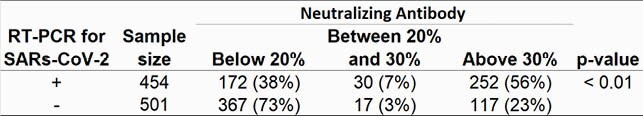

Figure 1. Scatter plot with Time between RT-PCR+ and neutralizing antibody (days) versus Neutralizing antibody levels.

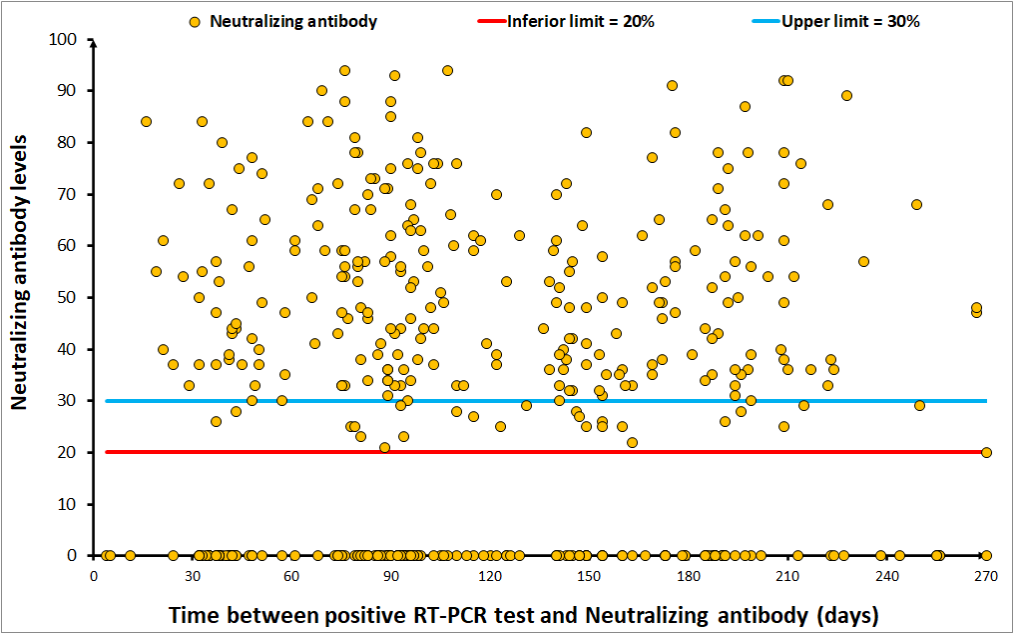

Table 2. Time between RT-PCR+ and neutralizing antibody (days) versus Neutralizing antibody levels.

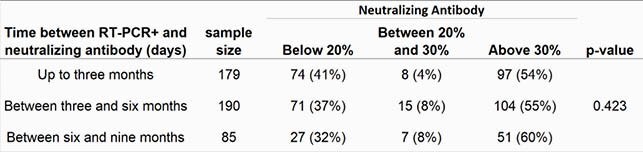

**Conclusion:**

This study found neutralizing activity of infection against SARS-CoV-2 in 63% RT-PCR+ individuals, but only in 26% in RT-PCR(-) players. Level of neutralizing antibody responses maintained stable until up to nine months after a RT-PCR+.

Figure 2. Percentage of soccer players at each antibody level (below 20%, between 20% and 30%, and above 30%) versus time between the positive RT-PCR test and neutralizing antibody test (days).

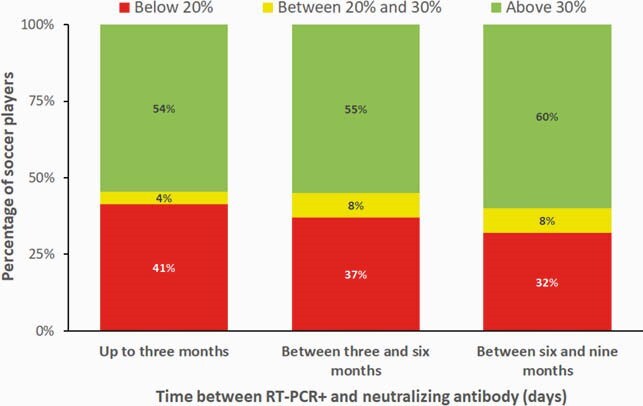

**Disclosures:**

**All Authors**: No reported disclosures

